# The Unphosphorylated EIIA^Ntr^ Protein Represses the Synthesis of Alkylresorcinols in *Azotobacter vinelandii*

**DOI:** 10.1371/journal.pone.0117184

**Published:** 2015-02-02

**Authors:** Luis Felipe Muriel-Millán, Soledad Moreno, Yanet Romero, Leidy Patricia Bedoya-Pérez, Miguel Castañeda, Daniel Segura, Guadalupe Espín

**Affiliations:** 1 Departamento de Microbiología Molecular, Instituto de Biotecnología, Universidad Nacional Autónoma de México, Cuernavaca, Morelos, México; 2 Centro de Investigaciones en Ciencias Microbiológicas, Instituto de Ciencias, Benemérita Universidad Autónoma de Puebla, Puebla, México

## Abstract

Upon encystment induction, *Azotobacter vinelandii* produces the phenolic lipids alkylresorcinols (ARs) that are structural components of the cysts. The enzymes responsible for the ARs synthesis are encoded in the *arsABCD* operon, whose expression is activated by ArpR. The transcription of *arpR* is initiated from an RpoS dependent promoter. The nitrogen-related phosphotransferase system (PTS^Ntr^) is a global regulatory system present in Gram negative bacteria. It comprises the EI^Ntr^, NPr and EIIA^Ntr^ proteins encoded by *ptsP*, *ptsO* and *ptsN* genes respectively. These proteins participate in a phosphoryl-group transfer from phosphoenolpyruvate to protein EIIA^Ntr^ via the phosphotransferases EI^Ntr^ and NPr. In *A. vinelandii*, the non-phosphorylated form of EIIA^Ntr^ was previously shown to repress the synthesis of poly-ß-hydroxybutyrate. In this work, we show that PTS^Ntr^ also regulates the synthesis of ARs. In a strain that carries unphosphorylated EIIA^Ntr^, the expression of *arpR* was reduced, while synthesis of ARs and transcription of *arsA* were almost abrogated. The expression of *arpR* from an RpoS-independent promoter in this strain restored the ARs synthesis. Taken together these results indicate that unphosphorylated EIIA^Ntr^ negatively affects activation of *arpR* transcription by RpoS.

## Introduction

*Azotobacter vinelandii* is a soil bacterium that undergoes a differentiation process resulting in the formation of a desiccation resistant cyst. A mature cyst consists of a contracted cell, known as the central body, which is surrounded by a capsule made up of a laminated outer layer called the exine and an inner layer called the intine [1]. The polysaccharide alginate is a major component of the capsule layers. Other components of the cysts are the reserve polyester poly-ß-hydroxybutyrate (PHB), that is present in the central body forming large granules, and the phenolic lipids alkylresorcinols (ARs), which replace the membrane phospholipids in the cyst and are also components of the exine [2]. Encystment can be induced by transferring log-phase vegetative cells to Burk’s minimal medium with either *n*-butanol or ß-hydroxybutyrate as the sole carbon source [3].

ARs play a structural role in the cyst, and strains carrying mutations in ARs biosynthetic genes produce cysts with a defective exine [4]. The *arsABCD* gene cluster encodes the enzymes that synthesize these lipids [5]. These genes are specifically expressed in encystment induction medium [4]. The transcriptional activator ArpR positively regulates transcription of the *arsABCD* operon, by direct binding to the *arsA* promoter region [6]. The mutational inactivation of *rpoS* impairs ARs synthesis [7] because this sigma factor is needed for the transcription of *arpR* [6].

The *ptsP*, *ptsO* and *ptsN* genes encode EI^Ntr^, NPr and EIIA^Ntr^ proteins, respectively, that are components of the nitrogen-related phosphotransferase system (PTS^Ntr^), which is homologous to the carbohydrate transport PTS. The PTS^Ntr^ proteins participate in a phosphoryl transfer chain from phosphoenolpyruvate, where EIIA^Ntr^ appears to be the terminal phosphoryl acceptor [8]. The PTS^Ntr^ regulates a wide variety of processes in bacteria; in *Legionella pneumophila*, a *ptsP* mutation, negatively affected its virulence in guinea pigs [9]; in *Rhizobium* species, the PTS^Ntr^ is associated to melanin synthesis, nitrogen fixation and regulation of ABC transport activation [10,11]. In *Escherichia coli*, the EIIA^Ntr^ protein controls the potassium transport by interacting with the Trk transporter subunit TrkA and the sensor kinase KdpD (that controls the expression of high affinity potassium transporter system KdpFABC) [12,13]. The response of *E*. *coli* to phosphate starvation is also activated by EIIA^Ntr^ due to an interaction with the sensor kinase PhoR [14].

In the *A*. *vinelandii* UW136, the non-phosphorylated form of EIIA^Ntr^ was shown to impair PHB production, by exerting a negative effect on expression of *phbR*, the gene encoding the transcriptional activator of the PHB biosynthetic operon *phbBAC* [15].

In this work we report the effect of mutations in the genes coding for the proteins of the PTS^Ntr^ on alkylresorcinol synthesis and show that the non-phosphorylated EIIA^Ntr^ protein has a negative effect on the transcriptional activation of *arpR* by RpoS.

## Materials and Methods

### Bacterial strains, plasmids and growth conditions

Bacterial strains and plasmids used are listed in S1 Table. *A*. *vinelandii* was cultured at 30°C in Burk’s nitrogen-free salts medium [16] supplemented with 2% sucrose (BS) for vegetative growth or 0.2% *n*-butanol (BBOH) for encystment induction. For determination of β-glucuronidase activity of transcriptional *phbR-gusA* and *phbB-gusA* fusions, the cells were grown in peptone yeast medium supplemented with 2% sucrose (PY). Liquid cultures were carried out in 250-mL or 125-ml flasks containing 50 or 25 ml of medium, respectively, in a rotatory shaker at 200 rpm and 30°C. Inocula for all experiments were grown on BS, washed three times with sterile 10mM MgSO_4_, and transferred to BBOH medium.

*E*. *coli* strain DH5α was grown in Luria-Bertani medium (LB) at 37°C. Transformation of *A*. *vinelandii* were carried out as previously described [16].

### Nucleic acid procedures

DNA purification and cloning procedures were carried out as previously described [17]. Total RNA extraction was performed as reported by Barry et al. [18]. DNA sequencing was done with a Perkin Elmer/Applied Biosystems DNA Sequencer. The sequences of oligonucleotides used in this work are described in the S2 Table.

### Constructions of transcriptional and translational fusions of *arpR* and *arsA* with *gusA* reporter

The pUMATc plasmid [19] was digested with *EcoRI* and *HindIII* to clone the *gusA* reporter gene obtained from pAHFUTs-Tc [20], resulting in the plasmid pUMATcgusAT.

The plasmids pUMATcgusAT and pUMATcgusAPT [19], unable to replicate in *A*. *vinelandii* and used for transcriptional and translational fusions, respectively, were digested with *SacI* and *KpnI* restriction enzymes to remove the tetracycline cassette. The ends of the plasmids were made blunt by treatment with Klenow fragment and used for cloning a blunted *MluI* gentamicin cassette obtained from pBSL98 [21]. The new plasmids pLM2 and pLM3 (S1 Table) were used to construct the transcriptional and translational fusions, respectively.

For the construction of transcriptional *arsA-gusA* and *arpR-gusA* fusions, DNA fragments of 1.0 and 0.99 Kb, containing the promoter region of *arsA* and *arpR*, respectively, were amplified using the primers FwarsA and RvarsAtrans and FwarpR and RvarpRtrans (S2 Table). The fragments were gel-purified, digested with *XbaI* and *PstI* and ligated to *XbaI-PstI* pLM2 vector to construct the plasmids pLM4 (*arsA-gusA*) and pLM6 (*arpR-gusA*). These plasmids were digested with *NdeI* and *ScaI*, respectively, and used to transform *A*. *vinelandii* strains for the selection of transformants carrying transcriptional *arsA-gusA* or *arpR-gusA* fusions integrated into the *melA* gene by a double recombination event. Digestion of the plasmids was carried out in order to avoid the selection of strains with plasmids integrated into the chromosome generated by single recombination events. The *melA* gene has been previously used as a neutral site to introduce gene fusions [19]. These strains are described in S1 Table.

For the construction of translational *arsA´-´gusA* and *arpR´-´gusA* fusions, DNA fragments of 1.3 and 1.1 Kb (containing the promoter region, the 5’ untranslated region and the first five codons of each gene) were amplified with FwarsA and RvarsAtrad and FwarpR and RvarpRtrad primers for *arsA* and *arpR*, respectively. The PCR products were purified, digested with *XbaI* and *PstI* enzymes and ligated to *XbaI-PstI* pLM3 resulting in the plasmids pLM5 (*arsA´-´gusA*) and pLM7 (*arpR´-´gusA*). The plasmids pLM5 and pLM7 were digested with *NdeI* and *ScaI*, respectively, and used to transform *A*. *vinelandii* strains for the selection of the transformants carrying translational *arsA´-´gusA* or *arpR´-´gusA* fusions described in S1 Table. The presence of all fusions in the strains was confirmed by PCR analysis (data not shown).

### Construction of plasmid pB*pgyrA-arpR* to express *arpR* from RpoS-independent promoter

First, we constructed the plasmid pJET-p*gyrA* cloning a 0.3 Kb DNA fragment containing the promoter region of *gyrA* gene (p*gyrA*) into vector pJET1.2/blunt (Thermo Scientific). A DNA fragment of 1.0 Kb containing the encoding region of *arpR* was amplified using the oligonucleotides arpRFw2 and arpRRv2 (S2 Table) and cloned into pJET-p*gyrA* downstream and the same direction of p*gyrA*. The fusion p*gyrA-arpR* was excised by digestion with *BglII* enzyme, gel purified, made blunt and cloned into *SmaI*-digested plasmid pBBR1MCs-5 [22], resulting in the plasmid pBp*gyrA-arpR*, which was transferred by conjugation into strain UW136::pALA8a.

### Quantitative Real Time PCR (q-RT-PCR)

Expression levels of *arsA* and *arpR* was measured by qRT-PCR as previously reported [15]. The primers used for the assays (S2 Table) were as follows: arsA-RT-F and arsA-RT-R for *arsA*, arpR-RT-F and arpR-RT-R for *arpR*, and fw-gyrA and rev-gyrA for *gyrA*. The level of *gyrA* was used as internal control to normalize the results. All assays were performed in triplicate. The data was analyzed by the 2^-Δ,ΔCT^ method reported by Livak and Shmittgen [23].

### Determination of alkylresorcinol production

The production of ARs was measured as previously described [24]. Briefly, the lipids were extracted with acetone for 1h at room temperature. The acetone extract was removed, and a second extraction was done for 12 h at room temperature. The resulting extracts were mixed and used for spectrophotometric determination of alkylresorcinols by the use of Fast Blue B as previously described [24]. Orcinol was used as a standard. The protein content of the cells used for AR determination was measured by the method of Lowry et al [25].

### Quantification of β-glucuronidase activity

The β-glucuronidase activity was measured as described previously [26] from encystment-induced cells in BBOH medium harvested to 72 hours of incubation. 1 U corresponds to 1 nmol of p-nitrophenyl-β-D-glucuronide hydrolyzed per minute per mg of protein.

## Results

### Effect of *ptsP*, *ptsO* and *ptsN* mutations on ARs synthesis

Strain UW136 is unable to produce alginate due to an insertion within the *algU* gene [27] therefore this strain is unable to produce genuine mature cysts, but under encystment induction medium produces ARs [4].

**Fig 1:**
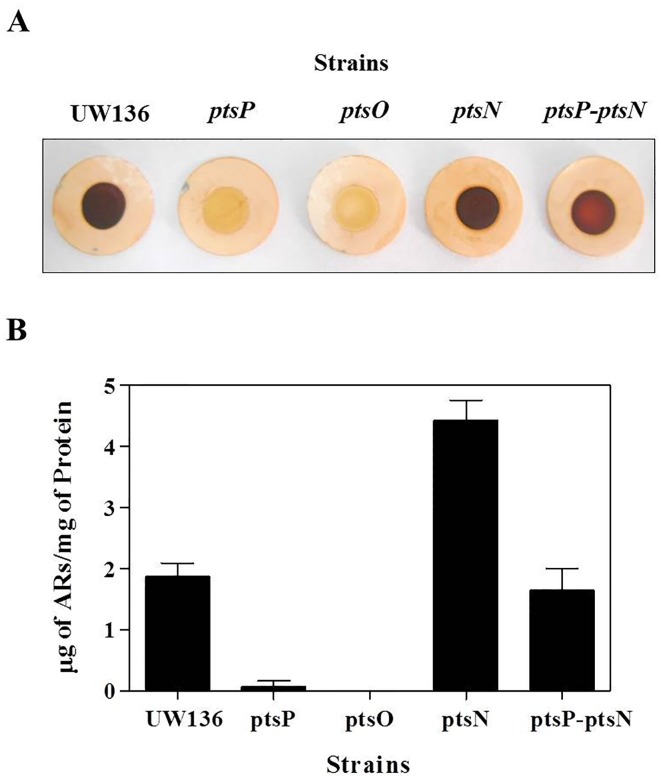
PTS^Ntr^ controls the ARs synthesis. Staining **(A)** and quantification **(B)** of alkylresorcinols produced by strains of *A*. *vinelandii* in BBOH medium to 120 hours of incubation. These data are mean of three independent experiments, error bars, SD.

To determine if PTS^Ntr^ is involved in the regulation of the ARs synthesis, we analyzed ARs production in encystment-induced cells of *pts* mutants, by staining these lipids with dye Fast Blue B [4]. The *ptsN* mutant and the UW136 wild type strain developed a red color indicative of ARs synthesis, while the *ptsP* and *ptsO* mutants remained white (Fig. 1A). The quantification of ARs production in these strains confirmed the observed phenotype in plates; no ARs were detected in the *ptsP* and *ptsO* mutants, while the *ptsN* mutant presented a significant increase in ARs production relative to the UW136 strain (Fig. 1B). According to the phosphorylation cascade proposed for the PTS^Ntr^ [15] the *ptsP* and *ptsO* inactivations are expected to impair the phosphorylation of EIIA^Ntr^, therefore, the unphosphorylated form of EIIA^Ntr^ could be involved in the negative effect observed on ARs synthesis. In agreement with this hypothesis, inactivation of *ptsN*, in the *ptsP* mutant background (*ptsP-ptsN* double mutant) restored the ARs synthesis (Fig. 1B).

### Effects of PTS^Ntr^ mutations on *arsA* expression

**Fig 2:**
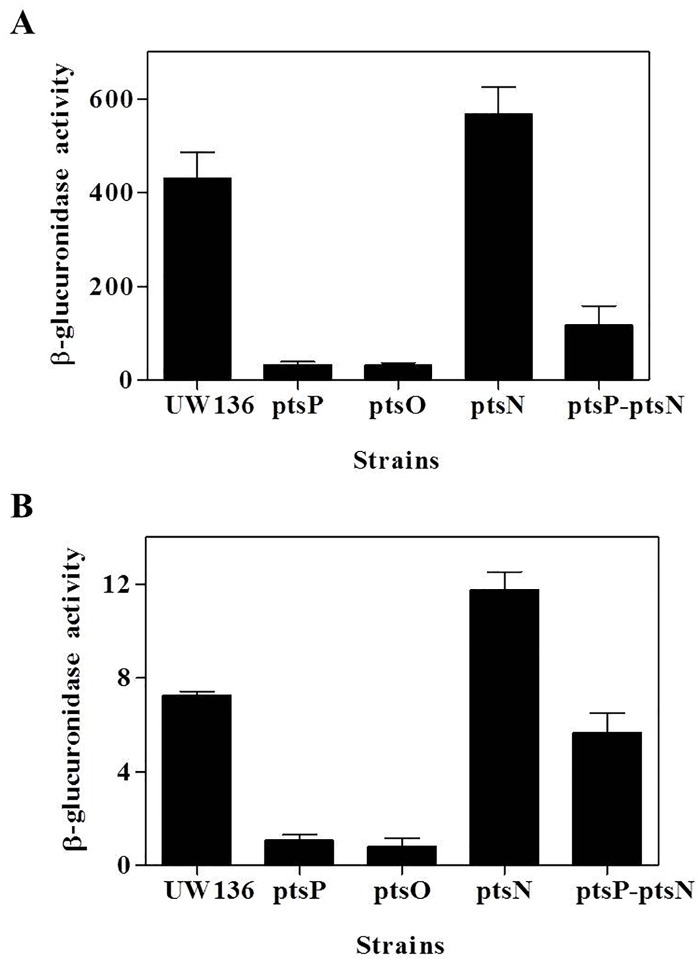
Effect of PTS^Ntr^ on *arsA* expression. β-glucuronidase activity in UW136 wild type strain and *pts* mutants carrying transcriptional *arsA-gusA* **(A)**, or translational *arsA´-´gusA* **(B)** gene fusions. The cells were grown for 72 h in BBOH medium at 30°C. The data represent the mean of three independent experiments. Error bars, SD.

In order to determine if PTS^Ntr^ affected ARs synthesis through an effect on *arsABCD* expression, transcriptional *arsA-gusA* and translational *arsA*´-´*gusA* gene fusions were used. The transcription and translation levels of *arsA* were determined by measuring β-glucuronidase activity in derivatives of the wild type UW136 strain and the *ptsP*, *ptsO*, *pstN* and *ptsP-ptsN* mutants carrying the gene fusions (S1 Table). We observed that *ptsP* and *ptsO* inactivations caused a similar decrease in the β-glucuronidase activity of both fusions (Fig. 2A and 2B), while in the *ptsN* mutant the activity increased in the transcriptional and translational fusions by 27% and 60% respectively. In the double mutant *ptsP-ptsN*, the β-glucuronidase activity of transcriptional fusion was partially restored, while the translational fusion showed a similar level to that observed in the wild type strain (Fig. 2A and 2B).

The level of *arsA* transcripts in the *pts* mutants was also evaluated by qRT-PCR. Table 1 shows that the *arsA* mRNA levels in *ptsP* and *ptsO* mutants were very low when compared to those observed in the wild type strain. In contrast, the *arsA* mRNA level was higher in the *ptsN* mutant and the double mutant *ptsP-ptsN* than in the UW136 strain. These results support the hypothesis that the non-phosphorylated form EIIA^Ntr^ negatively affects *arsA* expression at the transcriptional level.

**Table 1:** Relative mRNA levels of *arsA* and *arpR* in UW136 and *pts* mutants strains.

	Relative mRNA levels*	
Strain	arsA	arpR
UW136	1.0 ± 0.0	1.0 ± 0.0
PtsP	0.043 ± 0.007	0.021 ± 0.003
PtsO	0.099 ± 0.003	0.005 ± 0.003
PtsN	2.07 ± 0.24	2.7 ± 0.16
ptsP-ptsN	1.62 ± 0.10	1.3 ± 0.11

*The mRNA levels of *arsA* and *arpR* in *pts* mutants are relative to showed by UW136 strain, which are assumed to be 1.0. The values are the mean of two independent experiments.

### Effects of PTS^Ntr^ mutations on *arpR* expression

The results shown above suggest that PTS^Ntr^ controls the ARs synthesis trough the regulation of expression of *arsABCD*. We recently reported that ArpR, a LysR-type regulator, directly actives the transcription of *arsABCD* [6]. Therefore, the question of whether the PTS^Ntr^ affected the transcription of *arsA* through an effect on the *arpR* expression was raised.

**Fig 3:**
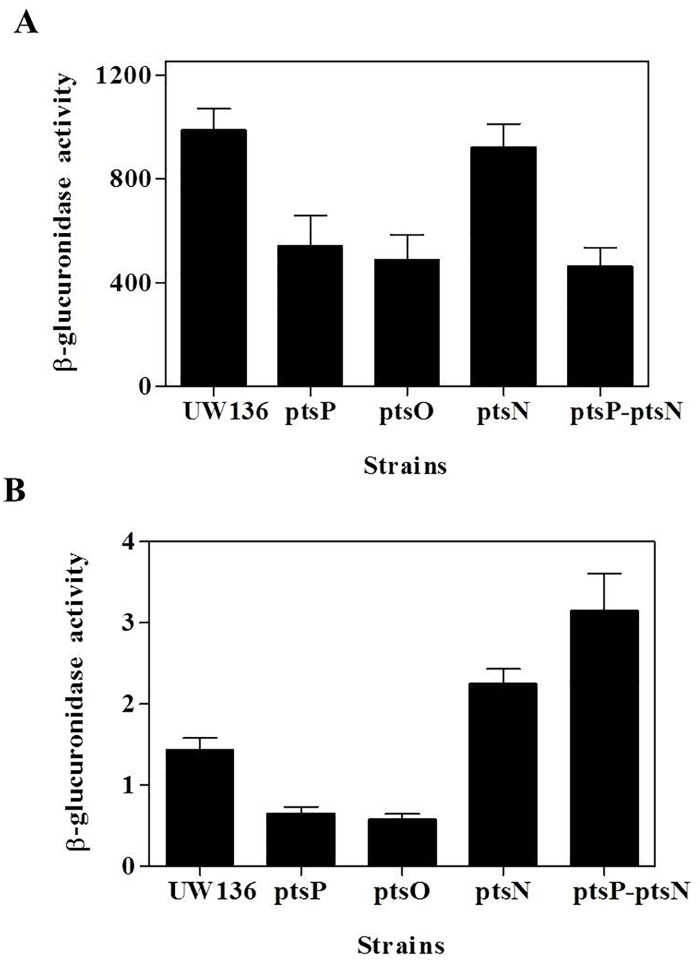
Effect of PTS^Ntr^ on *arpR* expression. β-glucuronidase activity in UW136 wild type strain and *pts* mutants carrying transcriptional *arpR-gusA* **(A)** or translational *arpR´-´gusA* **(B)** gene fusions. The cells were grown for 72 h in BBOH medium at 30°C. The data represent the mean of three independent experiments. Error bars, SD.

To study the effects of *pts* mutations on *arpR* expression, we used the UW136, *ptsP*, *ptsO*, *ptsN* and *ptsP-ptsN* strains carrying transcriptional and translational fusions of *arpR* (S1 Table). Fig. 3A shows that transcription of *arpR*, measured as β-glucuronidase activity, decreased about 40% in the *ptsP* and *ptsO* mutants relative to the wild type strain, while the *ptsN* inactivation had no affect on *arpR* transcription in the UW136, nor in the *ptsP* strains (Fig. 3A). The β-glucuronidase activity in the wild type and *pts* mutants carrying the translational *arpR´-´gusA* fusion (Fig. 3B), showed that the *ptsP* and *ptsO* mutations reduced about 50% the translation of *arpR*, as compared to the UW136 strain. In contrast, in the *ptsN* and *ptsP-ptsN* mutants a significant increase in the β-glucuronidase activity, relative to UW136 and *ptsP* strains respectively, was observed (Fig. 3B).

Using qRT-PCR, we found that the *ptsP* and *ptsO* mutations diminished the *arpR* mRNA level, while the *ptsN* inactivation increased it, in both the wild type UW136 and *ptsP* strains (Table 1). These results suggest that the non-phosphorylated EIIA^Ntr^ of the PTS^Ntr^ negatively controls *arpR* expression at the transcriptional and posttranscriptional levels.

### H68A mutation in the phosphorylation site of EIIA^Ntr^ impairs the transcription of *arpR*

**Fig 4:**
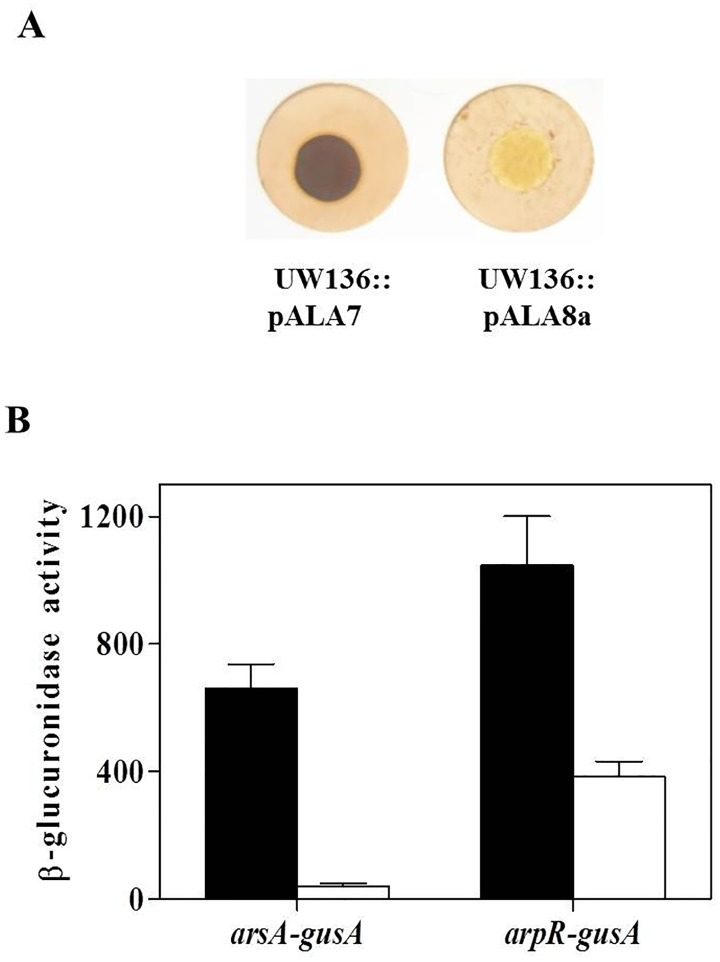
The unphosphorylated EIIA^Ntr^ negatively affects the ARs synthesis. Effect of H68A mutation on EIIA^Ntr^ on ARs production **(A)**, and activity of transcriptional *arsA-gusA* and *arpR-gusA* fusions **(B)**. The strains UW136::pALA7 (black bars) and UW136::pALA8a (white bars) carry an EIIA^Ntr^ and H68A EIIA^Ntr^, respectively. The cells were grown for 120 h for (A) and 72 h for (B) in BBOH medium at 30°C. The data represent the mean of three independent experiments. Error bars, SD.

The results presented above imply that the unphosphorylated form of EIIA^Ntr^ negatively controls the expression of *arpR*, affecting the transcription of *arsA* and, in turn, the synthesis of ARs. Thus, we tested the effect of a point mutation in *ptsN* (H68A), which produces a non-phosphorylatable EIIA^Ntr^, on ARs production and on transcription of *arpR* and *arsA*. For this experiment we used the strain UW136::pALA8a, which carries the *ptsN*-H68A mutation [15]. As shown in Fig. 4A, this strain showed a negative ARs production, similar to that observed in the *ptsP* and *ptsO* mutants (compare Figs. 1A and 4A). In contrast, the strain UW136::pALA7, which carries a wild type *ptsN* gene [15] presented a phenotype of ARs production, similar to UW136 wild type strain. As shown in Fig. 4B, the *ptsN*-H68A mutation almost abrogated the β-glucuronidase activity in the strain carrying the transcriptional *arsA-gusA* fusion, and reduced by 60% the activity of the *arpR-gusA* fusion relative to the strain UW136::pALA7. These results indicate that the unphosphorylated EIIA^Ntr^ protein represses the transcription of *arpR*.

### The negative effect of unphosphorylated EIIA^Ntr^ H68A on *arpR* transcription is through RpoS

**Fig 5:**
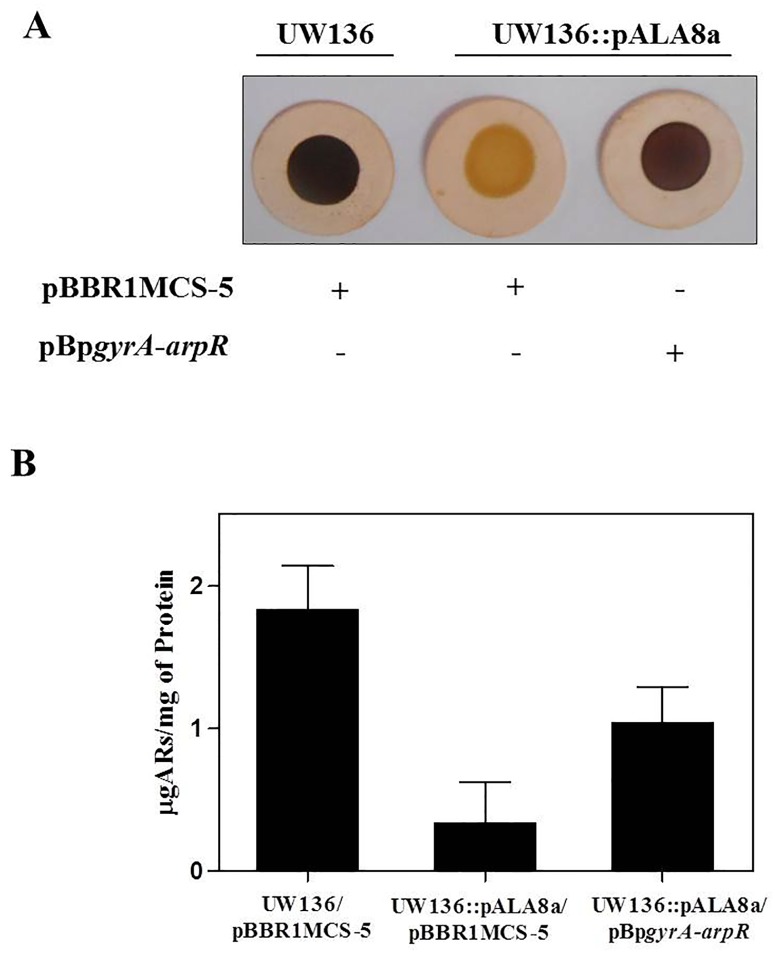
Effect of *arpR* expression from RpoS-independent promoter in the strain that carries the nonphosphorylatable EIIA^Ntr^ H68A protein. **(A)** Staining of ARs produced by UW136 and U136::pALA8a strains, transformed with plasmid PBpgyrA-arpR, carrying a constitutively expressed arpR gene or the empty plasmid pBBR1MCS-5 as negative control. **(B)** Quantification of ARs levels produced by the strains of the panel A. The data represent the mean of three independent experiments. Error bars, SD.

The data presented above indicate that transcription of *arpR* is negatively regulated by the unphosphorylated EIIA^Ntr^ protein. Since *arpR* transcription is dependent on RpoS [6], we wanted to determine if the unphosphorylated EIIA^Ntr^ affects *arpR* expression through this sigma factor. For this, we determined the capacity of AR synthesis in the strain UW136::pALA8a (expressing the unphosphorylatable EIIA^Ntr^) carrying the plasmid pBp*gyrA-arpR*, which expresses the *arpR* gene from an RpoS-independent promoter (*gyrA* promoter). As shown in the Fig. 5A, this strain was able to synthesize ARs in BBOH plates. In contrast, a negative phenotype of ARs production was shown by the strain UW136::pALA8a when was transformed with the empty plasmid pBBR1MCS-5. A similar effect was observed in BBOH liquid medium; the RpoS-independent expression of *arpR* increased the AR levels in the strain UW136::pALA8a (Fig. 5B). These results suggest that negative effect of unphosphorylated EIIA^Ntr^ on *arpR* expression is due to a negative effect on its transcriptional activation by RpoS.

## Discussion

The alkylresorcinols are exclusively synthesized during the encystment in *A*. *vinelandii*, since the expression of *arsABCD* operon is specifically activated in this condition [4]. Here, we identified that PTS^Ntr^ regulates the expression of ARs biosynthetic operon, through regulation of its transcriptional activator ArpR.

The PTS^Ntr^ is present in many bacterial genus and controls diverse physiological processes through the phosphorylation state of EIIA^Ntr^ [8]. Since mutations on *ptsP* or *ptsO* impair the phosphoryl-group transfer to EIIA^Ntr^, we hypothesized that absence of ARs synthesis in *ptsP* and *ptsO* mutants was mainly due to the presence of the unphosphorylated EIIA^Ntr^. This was confirmed by two approaches. First, the inactivation of *ptsN* was sufficient to restore the ARs levels in the *ptsP* mutant (Fig. 1B), and second, the strain that harbors an unphosphorylatable EIIA^Ntr^ H68A (which presents a replacement on the phosphorylation site histidine by an alanine) showed a negative ARs production phenotype (Fig. 4A).

Unexpectedly, in the *ptsP-ptsN* double mutant the ARs levels were lower than in the *ptsN* mutant (Fig. 1B). Additionally, the *ptsP* mutation produced a stronger negative effect on ARs production than the mutation producing an unphosphorylatable EIIA^Ntr^ H68A protein (compare Figs. 1B and 5B), suggesting a secondary regulatory role of EI^Ntr^ and/or NPr proteins on ARs synthesis independent of its role in the phosphorylation of EIIA^Ntr^. Additional experiments are necessary to validate this hypothesis.

The difference in ARs production between the *ptsN* and the *ptsP-ptsN* mutants could also be explained by the presence of an EIIA^Ntr^ paralog that partially complements the *ptsN* mutation. However a single *ptsN* gene was found in the *A*. *vinelandii* genome.

The transcription of *arsA* was reduced when EIIA^Ntr^ was present in its unphosphorylated form (Fig. 4B). Recently, we reported that both *arsABCD* and *arpR* transcription are directly activated by ArpR and acetoacetyl Coenzyme A (acetoacetyl-CoA) as coinducer [6]. Because the unphosphorylated EIIA^Ntr^ also reduced the *arpR* transcription (Fig. 4B), we concluded that the negative effect on *arsABCD* expression was due to a reduction of *arpR* expression. The negative effect of the EIIA^Ntr^ on expression of *arpR* could be explained by a reduction of the acetoacetyl-CoA pool. However this does not seems to be the case, since the presence of 5 and 50 μM of acetoacetyl-CoA did not restore the AR synthesis in *ptsP*, *ptsO* and *ptsN* H68A mutants (S1 Fig.). In contrast, an increase of ARs production phenotype dependent of acetoacetyl-CoA concentration was observed in the strains UW136, *ptsN* and *ptsP-ptsN* (S1 Fig.).

EIIA^Ntr^ has been shown to indirectly regulate the expression of several genes. For example, in *E*. *coli*, the interactions between EIIA^Ntr^ and kinase sensors KdpD and PhoR, increase the phosphorylation of response regulators KdpE and PhoB, resulting in increased expression of *kdpFABC* and the *pho* regulon, respectively [13,14]. Another interesting example is present in *Salmonella*, where EIIA^Ntr^ interacts with the SsrB response regulator, reducing the expression of *Salmonella* pathogenicity island 2 (SPI-2) [28]. Additionally, a relationship between EIIA^Ntr^ and the activity of sigma factors RpoS and RpoD has been previously described in *E*. *coli* [29]. In the absence of EIIA^Ntr^ (in a *ptsN* mutant), the potassium levels increase (by derepression of activity of K^+^ Trk transporter) resulting in preferential binding of the core RNA polymerase to RpoS instead of RpoD, and therefore, affecting the transcription of sigma regulons [29]. Here, we found that in *A*. *vinelandii* the negative effect of the unphosphorylated EIIA^Ntr^ on *arpR* transcription is through RpoS, since the expression of *arpR* from an RpoS-independent promoter was sufficient to restore ARs synthesis in the presence of unphosphorylated EIIA^Ntr^ (Figs. 5A and 5B). Further evidence supporting the participation of RpoS in the regulation exerted by EIIA^Ntr^ includes previous results showing that transcription of *phbR*, the gene encoding the transcriptional activator of PHB, and transcription of promoter pB_2_ of *phbBAC* are also RpoS dependent [30,31] and repressed by unphosphorylated EIIA^Ntr^ [15]. We carried out additional experiments to confirm the negative effect of unphosphorylated EIIA^Ntr^ protein on the *phbB* and *phbR* RpoS-dependent promoters (S2A and S2B Fig.). Indeed, the β-glucuronidase activity of transcriptional *phbR-gusA* and *phbB-gusA* fusions is reduced in the *ptsP* mutant (S2A and S2B Fig.). The mechanism by which the nonphosphorylated EIIA^Ntr^ affects the RpoS activity in *A*. *vinelandii* remains to be elucidated.

Nonphosphorylated EIIA^Ntr^ also seems to control the expression of *arpR* at a posttranscriptional level since the *ptsN* mutation increased the activity of the translational *arpR-gusA* fusion in the wild type and *ptsP* strains (Fig. 3B). Additionally, mutations of *ptsP* and *ptsO* diminished about twofold the activity of the transcriptional *arpR-gusA* fusion (Fig. 3A), while the *arpR* mRNA levels, measured by qRT-PCR, were even lower in the *ptsP* and *ptsO* mutants (Table 1). A similar effect was shown on the expression of *ilvBN* in *E*. *coli*, where a *ptsN* mutation reduced about 50% the activity of a transcriptional *ilvB-lacZ* fusion, while the *ilvB* mRNA levels (detected by RT-PCR) were more drastically reduced [32]. The mechanism by which nonphosphorylated EIIA^Ntr^ negatively affects the *arpR* expression at posttranscriptional level remains to be determined. However, as the translational *arpR* fusion contains the 5’ untranslated region of *arpR* mRNA (including the Shine-Dalgarno sequence), this mechanism could be related to a reduction of mRNA stability and/or to a blockage of translation.

**Fig 6:**
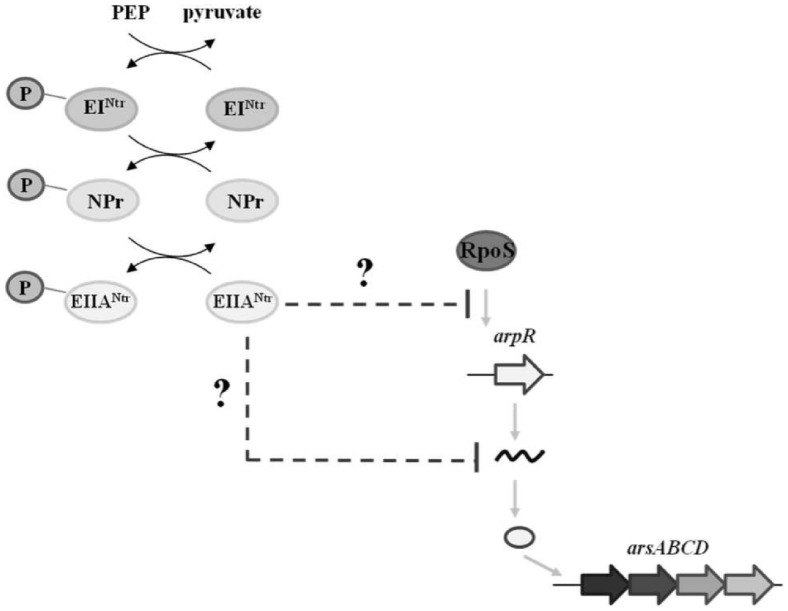
Model for the control of ARs synthesis by PTS^Ntr^ in *A*. *vinelandii*. The EIIA^Ntr^ protein in its unphosphorylated form represses the *arpR* expression both transcriptional (RpoS activity) and posttranscriptional levels. The dashed lines and gray arrows indicate negative effect and activation, respectively. PEP: Phosphoenolpyruvate, P: Phosphoryl group.

In summary, a regulatory model for the control of ARs synthesis by PTS^Ntr^ is proposed (Fig. 6). The EIIA^Ntr^ protein in its nonphosphorylated state inhibits the activation of the transcription of *arpR* by RpoS. The repression of *arpR* expression impairs the transcriptional activation of biosynthetic *arsABCD* operon. Additionally, EIIA^Ntr^ negatively affects the *arpR* mRNA levels by an unknown mechanism. The elucidation of the molecular mechanisms that link PTS^Ntr^ with RpoS and posttranscriptional regulation of *arpR* will allow us understand the role of PTS^Ntr^ in *A*. *vinelandii*.

## Supporting Information

S1 Fig Effect of acetoacetyl-CoA on ARs synthesis in *pts* mutant strains of *A*. *vinelandii*. The strains were grown in BBOH medium in absence or presence of 5 and 50 μM acetoacetyl-CoA (coinducer) for 72 h at 30°C.
(TIF)

S2 Fig Effect of the *ptsP* mutation on transcription of RpoS-dependent *phbR* and *phbB* genes. β-glucuronidase activity of transcriptional *phbR-gusA* **(A)** and *phbB-gusA* **(B)** fusions in UW136 and *ptsP* strains. The cells were grown in PY solid medium for 48 h at 30°C. The data represent the mean of two independent experiments. Error bars, SD.
(TIF)

S1 Table Strains and plasmids used in this work. (DOCX)

S2 Table Oligonucleotides used in this work. (DOCX)
