# Supplementary figures and images for: The Unphosphorylated EIIANtr Protein Represses the Synthesis of Alkylresorcinols in Azotobacter vinelandii
Source: PLoS One. 2015 Feb 2;10(2):e0117184. doi: 10.1371/journal.pone.0117184 (PMC4314083; doi:10.1371/journal.pone.0117184)

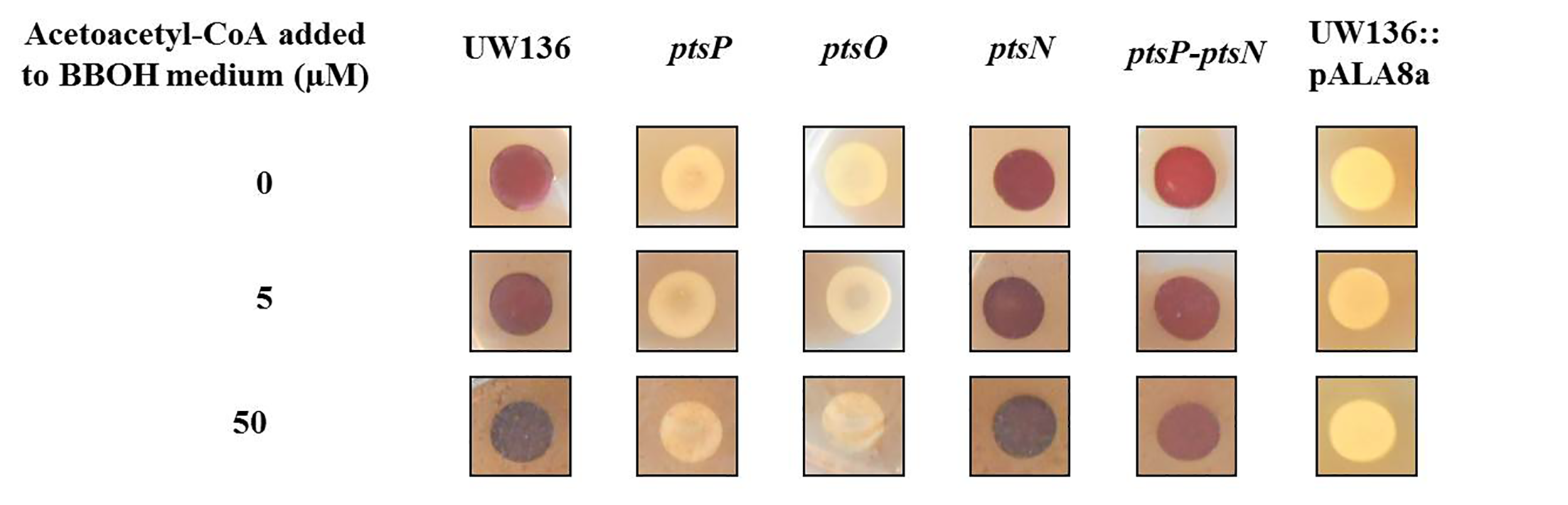

Supplement: S1 Fig — The strains were grown in BBOH medium in absence or presence of 5 and 50 μM acetoacetyl-CoA (coinducer) for 72 h at 30°C. (TIF) [file pone.0117184.s001.tif]

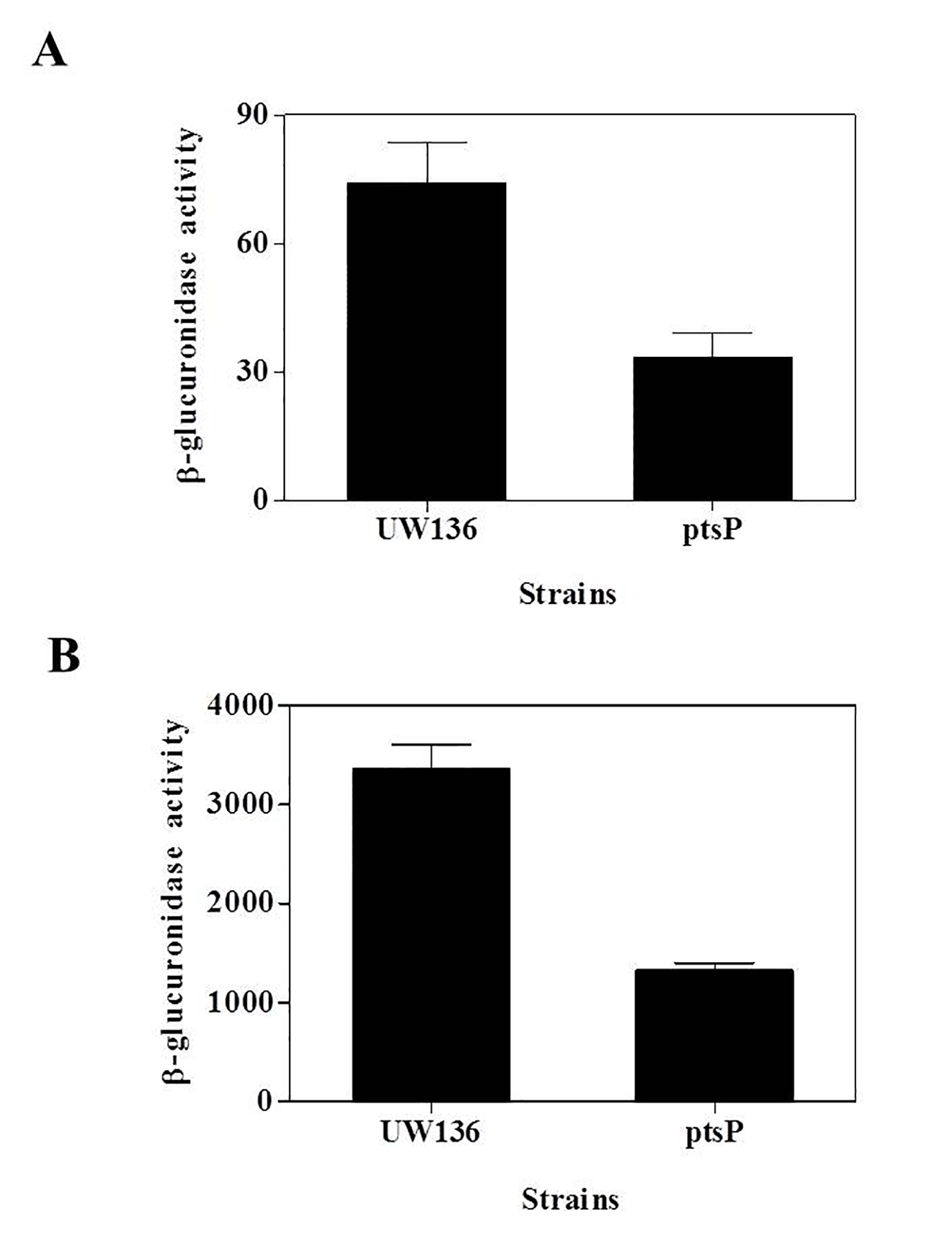

Supplement: S2 Fig — β-glucuronidase activity of transcriptional phbR-gusA (A) and phbB-gusA (B) fusions in UW136 and ptsP strains. The cells were grown in PY solid medium for 48 h at 30°C. The data represent the mean of two independent experiments. Error bars, SD. (TIF) [file pone.0117184.s002.tif]
