# Supplementary material for: The Unphosphorylated EIIANtr Protein Represses the Synthesis of Alkylresorcinols in Azotobacter vinelandii
Source: PLoS One. 2015 Feb 2;10(2):e0117184. doi: 10.1371/journal.pone.0117184 (PMC4314083; doi:10.1371/journal.pone.0117184)
Supplement: S1 Table — (DOCX) [file pone.0117184.s003.docx]

**Table S1.** Strains and plasmids used in this work

| **Strain** | **Description** | **Reference** |
| --- | --- | --- |
| *A. vinelandii* |  |  |
| UW136 | Natural mutation in *algU*, Nal^r^, Rif^r^ | [27] |
| RN4 | UW136 with a *ptsN*::Km mutation | [15] |
| RN6 | UW136 with a *ptsO*::Sp mutation | [15] |
| LSW1 | UW136 with a *ptsP*::Tc mutation | This work |
| LSW2 | UW136 with a *ptsN*::Sp mutation | This work |
| LSW3 | LSW1 with a *ptsN*::Sp mutation | This work |
| UW136::pALA7 | UW136 with pALA7 co-integrated expressing a phosphorylatable EIIA^Ntr^ | [15] |
| UW136::pALA8a | UW136 with pALA8 co-integrated expressing a nonphosphorylatable EIIA^Ntr^ H68A | [15] |
| LMW3 | UW136 carrying an *arsA-gusA* transcriptional fusion | This work |
| LMW4 | LSW1 carrying an *arsA-gusA* transcriptional fusion | This work |
| LMW5 | RN6 carrying an *arsA-gusA* transcriptional fusion | This work |
| LMW6 | RN4 carrying an *arsA-gusA* transcriptional fusion | This work |
| LMW7 | LSW3 carrying an *arsA-gusA* transcriptional fusion | This work |
| LMW8 | UW136::pALA7 carrying an *arsA-gusA* transcriptional fusion | This work |
| LMW9 | UW136::pALA8a carrying an *arsA-gusA* transcriptional fusion | This work |
| LMW10 | UW136 carrying an *arsA´-´gusA* translational fusion | This work |
| LMW11 | LSW1 carrying an *arsA´-´gusA* translational fusion | This work |
| LMW12 | RN6 carrying an *arsA´-´gusA* translational fusion | This work |
| LMW13 | RN4 carrying an *arsA´-´gusA* translational fusion | This work |
| LMW14 | LSW3 carrying an *arsA´-´gusA* translational fusion | This work |
| LMW15 | UW136 carrying an *arpR-gusA* transcriptional fusion | This work |
| LMW16 | LSW1 carrying an *arpR-gusA* transcriptional fusion | This work |
| LMW17 | RN6 carrying an *arpR-gusA* transcriptional fusion | This work |
| LMW18 | RN4 carrying an *arpR-gusA* transcriptional fusion | This work |
| LMW19 | LSW3 carrying an *arpR-gusA* transcriptional fusion | This work |
| LMW20 | UW136::pALA7 carrying an *arpR-gusA* transcriptional fusion | This work |
| LMW21 | UW136::pALA8a carrying an *arpR-gusA* transcriptional fusion | This work |
| LMW22 | UW136 carrying an *arpR´-´gusA* translational fusion | This work |
| LMW23 | LSW1 carrying an *arpR´-´gusA* translational fusion | This work |
| LMW24 | RN6 carrying an *arpR´-´gusA* translational fusion | This work |
| LMW25 | RN4 carrying an *arpR´-´gusA* translational fusion | This work |
| LMW26 | LSW3 carrying an *arpR´-´gusA* translational fusion | This work |
| UW136::pALA8a/pB*pgyrA-arpR* | UW136::pALA8a carrying pBp*gyrA-arpR* for *arpR* expression from an RpoS-independent promoter | This work |
| UW136/pBBR1MCS-5 | UW136 carrying empty plasmid pBBR1MCS-5 | This work |
| UW136::pALA8a/pBBR1MCS-5 | UW136::pALA8a carrying empty plasmid pBBR1MCS-5 | This work |
| *E. coli* |  |  |
| DH5α | *supE*44 Δ*lac*U169 *hsd*R17 *recA1 endA1 gyrA96 relA* | [17] |
| **Plasmids** |  |  |
| pBBR1MCS-5 | Cloning vector Gm^r^ | [22] |
| pBSL98 | Vector used to obtain Gm^r^ cassette | [21] |
| pUMATcgusAPT | Vector with the *gusA* gene for translational fusions | [19] |
| pUMATcgusAT | Vector with the *gusA* gene for transcriptional fusions | This work |
| pJET1.2 | Cloning vector | Thermo Scientific |
| pJET*pgyrA* | pJET1.2 derivative carrying promoter region of *gyrA* | This work |
| pLM2 | pUMATcgusAT Gm^r^ | This work |
| pLM3 | pUMATcgusAPT Gm^r^ | This work |
| pLM4 | pLM2 derivative carrying an *arsA*-*gusA* transcriptional fusion | This work |
| pLM5 | pLM3 trans derivative carrying an *arsA´-´gusA* translational fusion | This work |
| pLM6 | pLM2 trans derivative carrying an *arpR*-*gusA* transcriptional fusion | This work |
| pLM7 | pLM3 trans derivative carrying an *arpR´-´gusA* translational fusion | This work |
| pLM8 | pBBR1MCS-5 derivative carrying a fusion of *gyrA* promoter with *arpR* gene | This work |
