# Supplementary material for: The Unphosphorylated EIIANtr Protein Represses the Synthesis of Alkylresorcinols in Azotobacter vinelandii
Source: PLoS One. 2015 Feb 2;10(2):e0117184. doi: 10.1371/journal.pone.0117184 (PMC4314083; doi:10.1371/journal.pone.0117184)
Supplement: S2 Table — (DOCX) [file pone.0117184.s004.docx]

**Table S2. Oligonucleotides used in this study**

| **Name** | **Sequence** | **Reference** |
| --- | --- | --- |
| FwarsA | CATTCTAGAGTCTGTGCTGATCCATTGAT | This work |
| RvarsAtrans | CATCTGCAGACATGCTTGGTTTTCACACT | This work |
| RvarsAtrad | CATCTGCAGAATATGACTAGCCATTTGATC | This work |
| FwarpR | CATTCTAGAGATAGACGAAGCATCAGCAT | This work |
| RvarpRtrans | CATCTGCAGGCTGTTGGTTCCACGTCT | This work |
| RvarpRtrad | CATCTGCAGGTTCAGGGTATCCATATGG | This work |
| arpRFw2 | GATGGATGATCGCTTTTTG | This work |
| arpRRv2 | AAGTTTTCCTGGAACACTGC | This work |
| pgyrAupSacI | CGGAGCTCCCCATCGAAGAGCGTGAC | This work |
| pgyrAdwBamHI | CCGGATCCGTCAGCAGTGTCCTG | This work |
| arsA-RT-F | CACCCTCGTCAATCTGCTC | [4] |
| arsA-RT-R | GATCCTGGTCGAAGACCTTG | [4] |
| arpR-RT-F | CTTCCCCTGCTGGCACTC | [5] |
| arpR-RT-R | CGTTCCTGGAGTTCTTCGAG | [5] |
| Fw-gyrA | CCAGCAAGGGCAAGGTCTA | [15] |
| Rv-gyrA | TCGTCCAGCGGCAACAGGT | [15] |
